# Radiotherapy Metastatic Prostate Cancer Cell Lines Treated with Gold Nanorods Modulate miRNA Signatures

**DOI:** 10.3390/ijms25052754

**Published:** 2024-02-27

**Authors:** Sílvia Soares, Fátima Aires, Armanda Monteiro, Gabriela Pinto, Isabel Faria, Goreti Sales, Miguel A. Correa-Duarte, Susana Guerreiro, Rúben Fernandes

**Affiliations:** 1 (i3S), Instituto de Investigação e Inovação em Saúde, 4200-135 Porto, Portugal; 2 FP-I3ID, Instituto de Investigação, Inovação e Desenvolvimento, FP-BHS, Biomedical and Health Sciences, Universidade Fernando Pessoa (UFP), 4249-004 Porto, Portugal; 3 CECLIN, Centro de Estudos Clínicos, Hospital Escola Fernando Pessoa, 4420-096 Gondomar, Portugal; 4 Faculty of Chemistry, University of Vigo, 36310 Vigo, Spain; 5 CEB, Centre of Biological Engineering, Minho University, 4710-057 Braga, Portugal; 6 Biomark@UC/CEB–Centre of Biological Engineering of Minho University, Department of Chemical Engineering, Faculty of Sciences and Technology, Coimbra University, 3030-790 Coimbra, Portugal; 7 Radiotherapy Service, São João Hospital Center, 4200-319 Porto, Portugal; 8 CINBIO, University of Vigo, 36310 Vigo, Spain; 9 Southern Galicia Institute of Health Research (IISGS), Biomedical Research Networking Center for Mental Health (CIBERSAM), 36310 Madrid, Spain; 10 Institute of Molecular Pathology and Immunology of the University of Porto-IPATIMUP, 4200-465 Porto, Portugal; 11 Department of Biomedicine, Biochemistry Unit, Faculty of Medicine, University of Porto, 4200-319 Porto, Portugal; 12 Faculty of Nutrition and Food Sciences, University of Porto, 4150-180 Porto, Portugal; 13 UFP@RISE, Rede de Investigação em Saúde, Universidade Fernando Pessoa, 4249-004 Porto, Portugal

**Keywords:** gold nanorods, radiotherapy, microRNAs, prostate cancer cell lines

## Abstract

MicroRNA (miRNA) modulation has been identified as a promising strategy for improving the response of human prostate cancer (PCa) to radiotherapy (RT). Studies have shown that mimics or inhibitors of miRNAs could modulate the sensitivity of PCa cells to RT. In addition, pegylated gold nanoparticles have been studied as a therapeutic approach to treat PCa cells and/or vehicles for carrying miRNAs to the inside of cells. Therefore, we evaluated the capacity of hypofractionated RT and pegylated gold nanorods (AuNPr-PEG) to modulate the miRNA signature on PCa cells. Thus, RT-qPCR was used to analyze miRNA-95, miRNA-106-5p, miRNA-145-5p, and miRNA-541-3p on three human metastatic prostate cell lines (PC3, DU145, and LNCaP) and one human prostate epithelial cell line (HprEpiC, a non-tumor cell line) with and without treatment. Our results showed that miRNA expression levels depend on cell type and the treatment combination applied using RT and AuNPr-PEG. In addition, cells pre-treated with AuNPr-PEG and submitted to 2.5 Gy per day for 3 days decreased the expression levels of miRNA-95, miRNA-106, miRNA-145, and miRNA-541-3p. In conclusion, PCa patients submitted to hypofractionated RT could receive personalized treatment based on their metastatic cellular miRNA signature, and AuNPr-PEG could be used to increase metastatic cell radiosensitivity.

## 1. Introduction

MicroRNAs (miRNAs) are small, non-coding RNA molecules that play a vital role in regulating gene expression, which is involved in hallmarks of cancer, such as sustaining proliferative signaling, evading growth suppressors, resisting cell death, activating invasion and metastasis, and inducing angiogenesis [1,2]. Dysregulation of miRNA expression has been linked to a variety of diseases, including cancer [2]. The alteration of miRNA expression could contribute to cancer development and progression. MiRNAs can act as oncogenes, promoting cell proliferation and survival, or as tumor suppressors, inhibiting these cellular processes [3]. There is growing interest in the use of miRNAs as potential diagnostic biomarkers or therapeutic targets for cancer treatment [4,5], such as miRNA inhibition or miRNA degradation, which could modulate target gene expression. A better understanding of the role of miRNAs in cancer could lead to the development of new treatments and improve patient outcomes. 

Prostate cancer (PCa) represents a significant health concern globally, ranking as one of the most diagnosed malignancies among men. Its epidemiology showcases considerable geographical and ethnic variations [6]. In developed regions such as North America, Northwestern Europe, and Australia, PCa holds a prominent position as the most prevalent cancer among males. Advancing age is a key risk factor, with many cases occurring in men over the age of 50. Additionally, there is evidence of a genetic predisposition to the disease, with a higher incidence among individuals with a family history of PCa. Ethnic disparities are noticeable, with African American men having a substantially higher risk of developing and dying from PCa compared to men of other racial or ethnic groups [7]. Screening practices, access to healthcare, lifestyle factors such as diet and physical activity, as well as evolving diagnostic criteria, contribute to the complex epidemiological landscape of PCa. Understanding these diverse factors is crucial for devising effective prevention, screening, and treatment strategies for this prevalent malignancy.

PCa cells exhibit diverse biological behaviors that contribute to the complexity of the disease. Their growth and proliferation can vary widely, ranging from slow-growing tumors with relatively low aggressiveness to aggressive forms that rapidly spread beyond the prostate gland [8]. One hallmark of PCa cells is their dependence on androgen hormones, such as testosterone, for growth and survival, which is why androgen deprivation therapy is a standard treatment approach [9]. However, some cancer cells can evolve mechanisms to bypass this reliance, leading to treatment resistance and disease progression. Additionally, PCa cells could metastasize, often spreading to nearby lymph nodes, bones, or other distant organs, which significantly impacts the prognosis and treatment options for patients [10,11]. Therapies are sometimes not effective enough. Therefore, understanding the intricate biological behaviors of PCa cells is crucial for developing targeted therapies and personalized treatment strategies aimed at effectively managing the disease and improving patient outcomes.

Surgery and precision radiotherapy (RT) are the main methods of radical treatment for PCa [12]. Employing a comprehensive strategy centered around RT has resulted in outstanding curative outcomes, concurrently mitigating treatment-related side effects over time [13]. Over the past two decades, the outcomes of RT have undergone remarkable improvement, largely attributed to advancements in highly precise and conformal RT techniques. However, the outcomes of therapy are not fully satisfactory. Responses to RT exhibit significant variability among individuals, with some patients displaying resistance to this treatment and consequent local recurrences. Radioresistence stands as a primary obstacle hindering the effectiveness of RT [14]. While several biological changes within tumor cells—such as alterations in tumor metabolism, cell cycle arrest, modifications in oncogenes and tumor suppressors, changes in the tumor microenvironment, regulation of autophagy, generation of cancer stem cells, and responses to DNA damage and repair mechanisms—have been implicated in radioresistance, the precise mechanisms underlying this resistance to radiation remain largely elusive [14].

MiRNAs have gained significant attention for their dual role as both predictors of RT response and potent radiosensitizers in cancer treatment. The differential expression of specific miRNAs in tumor tissues has been correlated with the response to RT, serving as potential biomarkers to predict treatment outcomes and guide personalized therapeutic strategies [15]. Additionally, certain miRNAs have been identified as potent radiosensitizers capable of modulating the radiosensitivity of cancer cells [16]. Therefore, the cell’s radiation response could modulate protein expression involved in DNA damage repair, cell cycle checkpoints, apoptosis, autophagy, and the epithelial–mesenchymal transition, which can affect cancer cells’ sensitivity to RT. Our group reviewed the literature about the efficacy of RT miRNA’s key pathways involved in radioresistance [17]. Therefore, miRNA-95 can also target the G2/M checkpoint; its overexpression could accelerate the progression through the G2/M phase. MiRNA-106b behaved as an oncogene and targeted the pro-apoptotic caspase-7. MiRNA-145-5p directly targets androgen receptors and is involved in DNA double-strand break damage (DSBs) repair. MiRNA-541-3p directly interacts by suppressing Heat shock protein 27 (HSP27) and increases the sensitivity of the cells to radiation by increasing apoptosis. Dysregulation of miRNA expression has been observed in PCa treated with RT, and it is thought that targeting specific miRNAs could potentially improve the effectiveness of RT or reduce its side effects [18,19]. There is ongoing research on the role of miRNAs in response to RT and their potential as therapeutic targets to improve the effectiveness of this treatment. 

Gold nanoparticles (AuNPs) have emerged as promising radiosensitizers in the range of cancer treatment [20]. Their unique properties, such as high atomic number and surface plasmon resonance, make them excellent candidates for enhancing the effects of RT. When these nanoparticles are introduced into cancer cells and irradiated, they enhance the radiation’s impact by enhancing the generation of reactive oxygen species, increasing DNA damage, and inducing cellular apoptosis [21]. Their ability to selectively accumulate in tumor cells while sparing non-tumor cells improves the therapeutic outcome of radiation therapy. AuNPs, available in several shapes such as spheres, rods, stars, shells, and cages, offer a diverse spectrum of properties that influence their effectiveness as radiosensitizers in cancer therapy [22,23]. The distinct shapes of these nanoparticles play a crucial role in their interaction with radiation and cellular components. Our previous study showed that PEGylated gold nanorods (AuNPr-PEG) decreased cellular viability, migration, and survival fraction in a dose-dependent manner, suggesting that AuNPr-PEG could be used as a potential radiosensitizer [22]. AuNPr-PEG had a strong absorption of near-infrared (NIR) light, which can be used to trigger photothermal therapy. This therapy involves using NIR light to heat the nanoparticles, causing thermal damage to the cancer cells. This synergistic effect of RT and photothermal therapy can enhance the overall efficacy of the treatment [23]. However, little is known about AuNP treatments and miRNA expression after hypofractionated RT.

Our aim was to study the effect of AuNPr-PEG treatment with RT on the modulation of miRNA signature expression. Therefore, miR-95, -106b-5p, -145-5p, and -541-3p will be studied in human metastatic prostate cancer (PCa) cell lines, PC3, DU145, LNCaP, and the normal prostate epithelial cell line (HPrEpiC). In our methodology, pre-treated cells with AuNPr-PEG will be irradiated with three fractions of 2.5 Gy to simulate a hypofractionated radiation regimen similar to the patient clinical protocol.

## 2. Results

AuNPr-PEG synthesis and functionalization were previously described and characterized by Soares, et al., 2022 [22]. The human metastatic cell lines from PCa were pre-treated for 24 h with 0.01 mM of AuNPr-PEG and submitted to a clinical radiation plan, daily fractionated ionizing radiation (2.5 Gy dose of ionizing radiation per day) over three days. After that, RNA was isolated at different time points, and miR-95, miR-106-5p, miR-145-5p, and miR-541-3p expression levels were quantified by RT-qPCR (see Section 4.4.).

**Figure 1:**
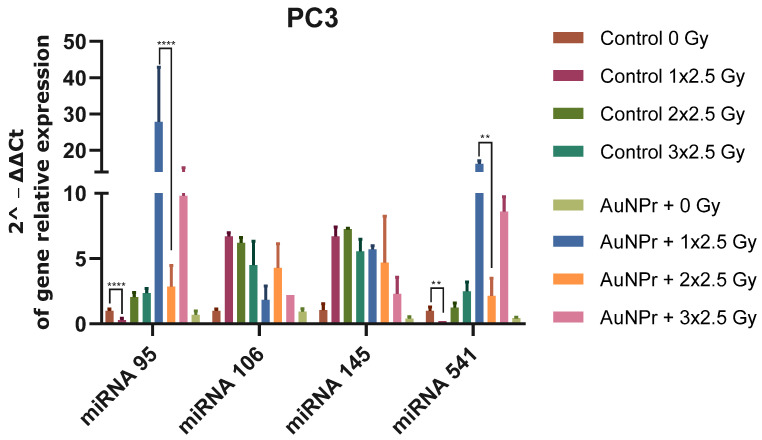
Graphical representation of miRNA expression levels in the PC3 prostate cancer cell line analyzed using RT-qPCR, compared to a control group that was not treated. The RNU6 housekeeping gene was used as a reference for normalization and relative expression was calculated using the ΔCT expression/ΔCT control ratio. The cells were irradiated in three doses of 2.5 Gy, with the first dose being 1 × 2.5 Gy, the second dose being 2 × 2.5 Gy, and the third dose being 3 × 2.5 Gy. ** *p* < 0.01; **** *p* < 0.0001. N = 2.

When PC3 cells were pre-treated with AuNPr-PEG without irradiation, an increase was observed in all evaluated miRNAs when compared to the control (non-treated and non-irradiated cells). However, the increase in miRNA-95 and miRNA 541-3p expression levels in cells was higher (Figure 1 and Figure S1). PC3 cells submitted to three doses of 2.5 Gy increased the expression levels of miR-95, miR-106-5p, miR-145-5p, and miR-541-3p when compared to non-irradiated cells. An inhibitory effect was observed when PC3 cells were pre-treated with AuNPr-PEG and submitted to three doses of 2.5 Gy, as the expression levels of miR-95, miR-106-5p, miR-145-5p, and miR-541-3p were decreased when compared to control cells without pre-treatment AuNP and submitted to three doses of 2.5 Gy (Figure 1).

**Figure 2:**
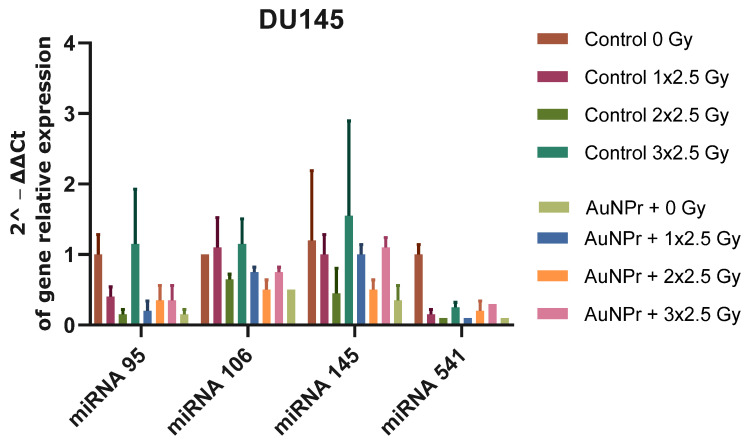
Graphical representation of miRNA expression levels in the DU145 cell line analyzed using RT-qPCR, compared to a control group that was not treated. The RNU6 housekeeping gene was used as a reference for normalization, and relative expression was calculated using the ΔCT expression/ΔCT control ratio. The cells were irradiated in three doses of 2.5 Gy, with the first dose being 1 × 2.5 Gy, the second dose being 2 × 2.5 Gy, and the third dose being 3 × 2.5 Gy. N = 2.

Figure 2 shows the results when Du145 cells were pre-treated with AuNPr-PEG without irradiation. A decrease in all miRNAs evaluated was observed when compared to the control (non-treated and non-irradiated cells); however, the decrease in miRNA-95 and miRNA 541-3p expression levels in cells is more significative. In Du145 cells submitted to three doses of 2.5 Gy, the expression levels of miR-95, miR-106-5p, and miR-145-5p were not altered; however, a significant decrease in miR-541-3p was observed when compared to non-irradiated cells. An inhibitory effect was observed when Du145 cells were pre-treated with AuNPr-PEG and submitted to three doses of 2.5 Gy, and the expression levels of miR-95, miR-106-5p, miR-145-5p, and miR-541-3p were decreased when compared to control cells without pre-treatment AuNP and submitted to three doses of 2.5 Gy.

**Figure 3:**
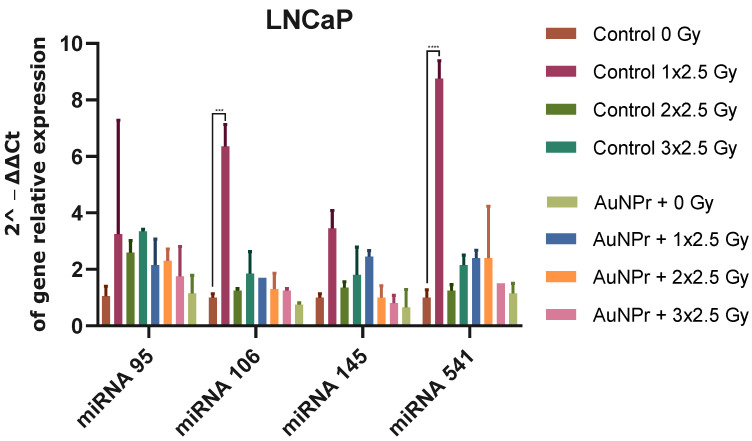
Graphical representation of miRNA expression levels in the LNCaP cell line analyzed using RT-qPCR, compared to a control group that was not treated. The RNU6 housekeeping gene was used as a reference for normalization and relative expression was calculated using the ΔCT expression/ΔCT control ratio. The cells were irradiated in three doses of 2.5 Gy, with the first dose being 1 × 2.5 Gy, the second dose being 2 × 2.5 Gy, and the third dose being 3 × 2.5 Gy. *** *p* < 0.001; **** *p* < 0.0001. N = 2.

Figure 3 shows the results when LNCaP cells were pre-treated with AuNPr-PEG without irradiation. An increase in all miRNAs evaluated was observed when compared to the control (non-treated and non-irradiated cells). LNCaP cells submitted to three doses of 2.5 Gy increased the expression levels of miR-95 and miR-541-3p more significantly than miR-106-5p and miR-145-5p when compared to non-irradiated cells. An inhibitory effect was observed when LNCaP cells were pre-treated with AuNPr-PEG and submitted to three doses of 2.5 Gy. The expression levels of miR-95, miR-106-5p, miR-145-5p, and miR-541-3p decreased when compared to control cells not pre-treated with AuNP and submitted to three doses of 2.5 Gy (Figure 3).

**Figure 4:**
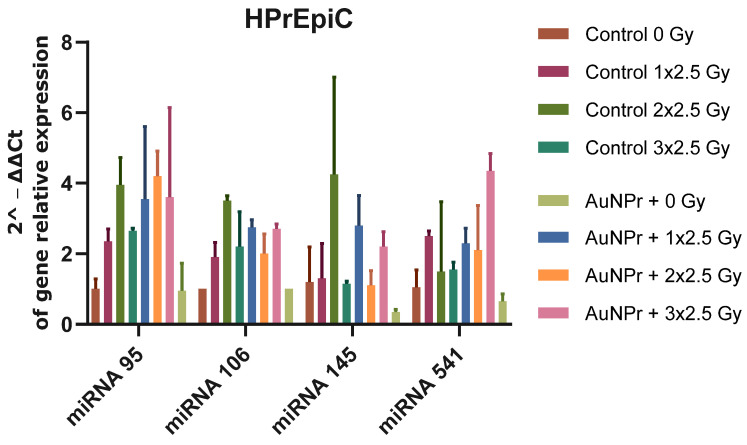
Graphical representation of miRNA expression levels in the HPrEpiC cell line isolated from normal human prostate tissue. MiRNA expression was analyzed using RT-qPCR and compared to a control group that was not treated. The RNU6 housekeeping gene was used as a reference for normalization and relative expression was calculated using the ΔCT expression/ΔCT control ratio. The cells were irradiated in three doses of 2.5 Gy, with the first dose being 1 × 2.5 Gy, the second dose being 2 × 2.5 Gy, and the third dose being 3 × 2.5 Gy. N = 2.

In addition, the miRNA expression in a non-tumor cell line, HPrEpiC, was studied. This is a cell line isolated from normal human prostate tissue (Figure 4 and Figure S4). HPrEpiC pre-treated with AuNPr-PEG increased the expression levels of all miRNAs studied when compared to non-treated cells. The cellular expression levels of miRNA-95 and miRNA-106 in HPrEpiC cells increased with RT, and no significant differences regarding miRNA-145 and miRNA-541-3p were found when compared to non-irradiated cells. In addition, when cells were submitted to RT and pre-treated with AuNPr-PEG, all miRNA studied decreased after three 2.5 Gy when compared to the control (control 3 × 2.5 Gy). 

## 3. Discussion

MiRNA is expected to account for 1–5% of the human genome and to interfere with at least 30% of the protein-coding genes [24,25]. Recent studies have found that miRNA expression can be altered in cancer cells after exposure to RT, which can affect the response of the cells to the treatment. Some miRNAs have been found to promote cell death in response to radiation, while others can protect the cells and make them more resistant to the treatment. Nearly 50% of PCa patients will suffer from radioresistance within five years due to the cell’s adaptation to RT, which causes radioresistance [26,27]. According to the literature, miRNA-95-5p, -106b-5p, 145-5p, and -541-3p were important in regulating gene expression of radiation response in PCa cells. To identify the PCa miRNA signature and to improve radiation response, the expression profiling of four miRNAs was evaluated in three metastatic PCa cell lines (PC3, DU145, and LNCaP) and one non-tumor prostate cell line (HPrEPiC). To the best of our knowledge, this is the first study to evaluate the effects of AuNPr-PEG treatment with RT on miRNA expression in PCa cell lines. This study applied an irradiation methodology of three doses of 2.5 Gy on cells, which makes it difficult to compare the results with the literature. Considering all the data, the findings of this study were compared with those reported in the literature. Although some of the results were contradictory to what is described in the literature, it is crucial to recognize that comparing studies that utilize different methodologies can be difficult. The review of the data is presented in Table 1.

Our study revealed a significant reduction in miRNA-95 expression in DU145 and LNCaP prostate cancer cells following exposure to a 2.5 Gy radiation dose. This decrease in miRNA-95 levels suggests a potential strategy to enhance the radiosensitivity of these cancer cells, making them more susceptible to radiation therapy. Moreover, our findings indicate that treatment with AuNPr-PEG in conjunction with radiation therapy further diminishes miRNA-95 expression. This outcome highlights the capability of AuNPr-PEG as an effective miRNA modulator, opening new avenues for targeted cancer therapy by manipulating miRNA levels to optimize therapeutic efficacy. Conversely, we observed an upregulation of miRNA-95 in PC3 prostate cancer cells and HPrEpiC non-tumor epithelial cells. The overexpression of miRNA-95 in these cell lines suggests a potential mechanism for enhanced radioresistance, particularly in the non-tumor HPrEpiC line, which may possess inherent protective responses against radiation-induced damage. This differential expression pattern underscores the complexity of miRNA regulation in response to radiation across different cell types and the potential for miRNA modulation to either sensitize or protect cells from radiation therapy. Our observations regarding the PC3 cell line are consistent with the existing literature, reinforcing the notion that miRNA-95 may play a role in modulating radioresistance in certain prostate cancer cells. However, the unique response of DU145 cells, as compared to previously reported findings, emphasizes the variability in miRNA-mediated responses to radiation among different cancer cell types [38].

The expression of MiRNA-106b-5p increased in PC3, LNCaP, and HPrEpiC cells, but only increased by a first and third fraction in the DU145 cell line, 10%, and 14%, respectively. MiRNA expression was also changed by AuNPr-PEG, which provided a reduction of miRNA-106b-5p after RT in PC3, DU145, and LNCaP cells. In relation to the non-tumor cell line, HPrEpiC, they exhibited an opposite performance with AuNPr-PEG treatment and their expression increased, inducing radioresistence.

Here, the results were the opposite, where an irradiation dose of 2.5 Gy seemed to increase mIRNA-145-5p expression in PC3 and LNCaP cells. Concerning the DU145 cell line, miRNa-145 was enhanced by only 55% in the third fraction of irradiation. In HPrEpiC cells, miRNA-145-5p only increased by a factor of three compared to non-irradiated cells on the second fraction. After applying AuNPr-PEG, miRNA expression was inverted in PC3 and LNCaP, which has no therapeutic benefit in this case. In DU145 cells, AuNPr-PEG increased miRNA-145-5p expression by only 6% on the second fraction compared to the non-treated group. In HPrEpiC cells, a two-fold increase occurred on the first and third fractions.

Furthermore, the results show a decrease in miRNA-541-3p expression after irradiation in DU145 cells, and an increase in LNCaP and HPrEpiC cells. In PC3 cells, these miRNAs were overexpressed in the second and third fractions. After AuNPr-PEG treatment, PC3 cells demonstrated a similar result, but miRNA-541-3p expression was more pronounced with nanoparticles. In addition, in HPrEpiC cells, AuNPr-PEG expressed an increase only in the second and third fractions of RT. However, AuNPr-PEG also influenced the reduction in miRNA expression in the DU145 and LNCaP cell lines.

Concerning their expression and therapeutic benefit, two main strategies can be selected for developing miRNA-based therapies: restoring lost miRNA function or silencing over-expressed miRNA [39]. These can be achieved using miRNA mimetics, synthetic double-stranded RNA molecules that mimic endogenous miRNAs. However, these have limited cellular uptake and stability issues. Another approach uses anti-miRs or antagomiRs, single-stranded RNAs that are chemically modified to improve binding, stability, and inhibition effect [39].

The differences between studies in the literature and our results could be related to the use of distinct methodologies of RT. Most of protocols of RT use a single dose per fraction. The RT treatment’s length could be another factor affecting cell-dependent miRNA expression levels.

In PCa, miRNAs-95 can also directly disrupt the G2/M checkpoint following radiation, which is overexpressed and can accelerate progression through the G2/M phase, contributing to radioresistence [38]. However, other studies show that the low expression of miRNA-95 after RT was related to the increase in radiosensitivity and promoted apoptosis [40,41,42]. Findings in lung and rectal cancer cell lines reported that miRNA-95 was found to be overexpressed in lung cells, but under-expressed in rectal cells [40,41,42].

Analogous to miRNA-95, the downregulation of miRNA-106b-5p appeared to enhance radiosensitivity. Studies in different cancer cells have demonstrated the role of miR-106b on p21 regulation after RT, which causes overrode radiation-induced cell cycle arrest and promotes proliferation [31,43,44]. In rectal cancer, the overexpression of miR-106b directly decreases the expression levels of p21 and mediates G1 growth arrest and cellular senescence [43,45]. In addition, studies showed that miRNA-106b behaved as an oncogene and targeted the pro-apoptotic caspase-7 [30,45,46]. Li et al. exhibited an opposite result, where miRNA-106b decreased their expression on LNCaP cells after being irradiated with a single dose of 6 Gy [31]. 

Another miRNA explored was miRNA-145-5p, which directly targets androgen receptors and is involved in DNA double-strand break damage (DSB) repair [33,34,47]. This miRNA was overexpressed in high-risk PCa patients after RT [48,49]. Studies have shown that overexpression of miRNA-145 can improve radiosensitivity by decreasing DNA DSBs and directly targeting oncogenes [33,34,50,51]. El Bezawy et al. related the overexpression of miRNA-145 with suppression of the activity of DNA (cytosine-5-)-methyltransferase 3 beta (DNMT3b) and adhesion molecules such as E-cadherin [32,36]. Wang et al. showed that microRNA-145 induces growth arrest in human PC3 cells [52]. This miRNA sensitized PC3 and LNCaP cells but was described as downregulated after irradiation in LNCaP cells [33]. 

Additionally, miRNA-541-3p was analyzed and, according to recent studies, this miRNA was found at low levels in PCa tissue, but when exposed to RT, it is overexpressed in PCa cells (PC3, DU145, and LNCaP). With the mimic approach, it was found that miRNA-541-3p directly interacts by suppressing Heat shock protein 27 (HSP27) and increases the sensitivity of the cells to radiation by increasing apoptosis and reducing β-catenin levels [37]. 

## 4. Materials and Methods

### 4.1. Reagents

Fetal bovine serum (FBS), phosphate-buffered saline (PBS), trypsin-EDTA, antibiotic antimycotic solution, thiol-polyethylene glycol-amine (SH-PEG-NH2), molecular weight 2kDa, trisodium citrate dehydrate (C_6_H_5_O_7_Na_3_·2H_2_O or NaCt), tetrachloroauric acid tetrahydrate (HAuCl_4_·4H_2_O, 99.99%), silver nitrate (AgNO_4_), and L-ascorbic acid, ≥99% were purchased from Sigma Aldrich^®^ LLC (St. Louis, MO, USA). Roswell Park Memorial Institute (RPMI-1640) media and Minimum Essential Medium (MEM) were purchased from Biowest^®^ (Nuaillé, France). QIAzol lysis reagent was purchased from QIAGEN Inc. (Valencia, CA, USA). Norgen’s MicroScript microRNA cDNA Synthesis Kit was purchased from Norgen BioteK (Thorold, ON, Canada).

### 4.2. Synthesis of Gold Nanorods 

AuNPr was produced according to a protocol developed by Scarabelli and coworkers, which involves growing a seed solution with AgNO_3_ and L-ascorbic acid [53]. After synthesis, the nanoparticles were PEGylated by adding SH-PEG-NH_2_ to the AuNPr solution and stirring for 24 h. The solution obtained was washed twice at 7500 rpm for 30 min and the pellet of PEGylated gold nanorods (AuNPr-PEG) was resuspended in water.

### 4.3. Cell Culture 

PC3, DU145, and LNCaP cell lines were provided by the Cancer Biology and Epigenetics Group at the Portuguese Oncology Institute of Porto, and human prostate Epithelial cells (HPrEpiC) were acquired from Innoprot (Innovative Technologies in Biological Systems, Derio, Spain). The PC3, LNCaP, and HPrEpiC cells were grown in RPMI-1640 media, while the DU145 cells were grown in MEM media, both supplemented with 10% FBS and 1% penicillin and streptomycin. The cells were cultured until they reached approximately 80% confluence and were then sub-cultured. All the experiments were incubated at 37ºC in a humid environment with 5% CO_2_.

### 4.4. Treatment Protocol 

**Figure 5:**
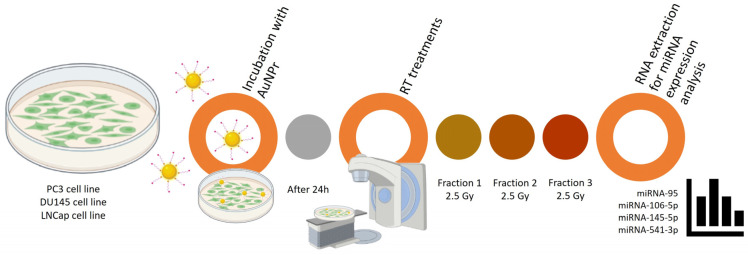
Representative scheme of the workflow for AuNPr-PEG and RT treatment in PC3, DU145, and LNCaP cell lines. Here, the protocol steps are summarized in fundamental sections from plating, treatment with AuNPr-PEG, treatment with radiotherapy (2.5 Gy/fraction), and extraction and analysis of the expression of miRNAs (miRNA-95, miRNA-106-5p, miRNA-145-5p, and miRNA-541-3p).

Depending on the cell line, cells were cultured in 6-well plates with 40,000 to 70,000 cells/mL until fully adhered (Figure 5). Then, cells were treated with 0.1 mM of AuNPr-PEG for 24 h. After, cells were washed to remove the excess of AuNPr-PEG, following one single irradiation of 2.5 Gy with a 6 MV photon beam repeated on three consecutive days until an accumulative dose of 7.5 Gy was reached. Cells were trypsinized on days 1, 2, and 3, and RNA was extracted for miRNA expression analysis. The control group included cells that received no treatment and were treated only with AuNPr-PEG with respective radiation doses.

### 4.5. RNA Isolation and Gene Expression

Total RNA was extracted from the treated cells using the Lab-Aid 824s Nucleic Acid Extraction System (Zeesan, Fujian, China) and the purity of the RNA was assessed using a spectrophotometer. The RNA was then reverse transcribed using Norgen’s MicroScript microRNA cDNA Synthesis Kit following the manufacturer’s instructions. DNA and RNA purity was evaluated by a 260/280 nm ratio. After, RNA samples were submitted to RT-qPCR using a SensiFAST™ SYBR^®^ No-ROX Meridian kit according to the manufacturer’s recommendation and applying specific primer sets specific to miR-95, -106b-5p, 145-5p, and -541-3p (Table 2). 

Threshold cycle (CT) values from each sample were plotted with four experimental replicates following the manufacturer’s procedure. The expression levels of the RNA were normalized to the expression of the RNU6 housekeeping gene and gene-relative expression was employed by the ΔCT expression/ΔCT control ratio.

### 4.6. Cell Irradiation|Irradiation Setup

**Figure 6:**
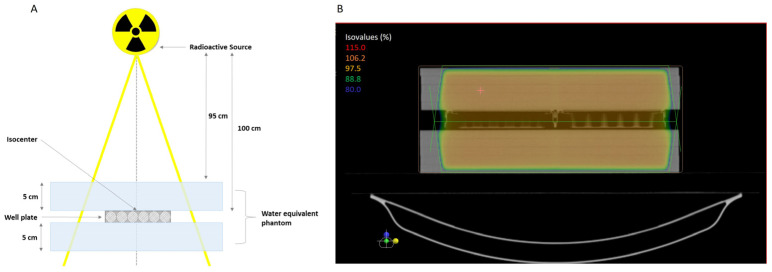
(**A**) Representative scheme of the standard irradiation setup to deliver 6 MV irradiation to the isocenter of the cell-well plate using a linear accelerator. The plate was plated between water-equivalent phantoms to simulate the tissues and depth. (**B**) Illustrative image of the treatment plan where the isodose lines are visible, surrounding the treatment area with at least 95% of the dose.

The setup of 6-well plates was put through computed tomography scans to obtain three-dimensional (3D) images. After, dosimetric planning was prepared using the software XIO-Release version 4.70.02, and the dose was prescribed to the isocenter using two fields (one anteroposterior and one posteroanterior) to provide a homogenous dose distribution. In addition, a plaque was incorporated by the 95% isodose line. The total dose applied was 7.5 Gy delivered in three fractions of 2.5 Gy using a 6 MV photon beam generated by a PRIMUS linear accelerator (Siemens). The control group did not receive radiation or AuNPr-PEG. A bolus of 5 cm thickness was placed on top and under the plates to simulate a biological structure and provide sufficient backscatter radiation to form an electric equilibrium. The irradiation setup is presented in Figure 6. 

### 4.7. Statistical Analysis

The data presented in this study are the mean and their standard deviations (SD). The data were analyzed using Prism 8.0 software. The differences between treatments were determined using two-way ANOVA with the Sidak multiple comparisons test. Results were considered statistically significant if the *p*-value was less than 0.05.

## 5. Conclusions

Overall, miRNA expression plays an important role in the RT response to cancer cells. Our preliminary study showed that cell types had different miRNA expression levels, and miRNA expression levels could be modulated by cell irradiation and AuNPr-PEG treatment. MiRNA expression levels exhibited variability based on the specific cell type and the treatment combinations utilized, including RT and AuNPr-PEG. Analyzing the outcomes, miRNA-106-5p and -541-3p demonstrate the potential to heighten the radiosensitivity of PC3 cells, whereas miRNA-95 and -106-5p exhibit the capability to enhance the radiosensitivity of DU145 and LNCAP cells. Conversely, the treatments promoted the radioprotection of HPrEpiC cells. These findings suggest that RT combined with AuNPr-PEG treatment could potentially manipulate the miRNA profile in PCa patients, potentially offering valuable insights for tailoring personalized RT approaches. Using the predictive value and radiosensitizing potential of miRNAs holds promise for optimizing treatment strategies, improving patient outcomes, and advancing precision medicine in cancer therapy. However, further research is needed to better understand the molecular pathways in regulating radiation-induced cellular responses, such as cell cycle arrest, cell proliferation inhibition, or cell death. In this sense, understanding how miRNA expression is affected by radiation can help to develop new strategies for improving the effectiveness of RT while reducing the side effects on normal cells.

## Figures and Tables

**Table 1:** MiRNA expression in radiation response in prostate cancer cell lines. With regard to the data presented from our study, the results of the 3rd day of the study, i.e., 3 × 2.5 Gy, were taken into account.

miRNA	Cell Line Used in the Literature	Function	Target	miRNA Expression after Irradiation		Functional Role	Therapeutic Strategy	References
				In the Literature (2–8 Gy)	Our Study (3 × 2.5 Gy)			
**hsa-miRNA-95**	PC3	-	SGPPI	↑ (6 Gy)	↑ PC3, LNCaP, HPrEPiC↓ DU145	RR	Antagomirs	[28,29]
**hsa-miRNA-106b**	LNCaP	OM	P21 P53 Caspase-7	↓ (6 Gy)	↑ PC3, LNCaP, HPrEPiC ↓ DU145	RR	Antagomirs	[30,31]
**hsa-miRNA-145**	LNCaP, PC3	TS	DNMT3b SPOP ZEB1	↑ (2 Gy)	↑ PC3, DU145, HPrEPiC ↓ LNCAP	RR	Mimicking	[32,33,34,35,36]
**hsa-miRNA-541-3p**	LNCaP, DU145, PC3, and PrEC	TS	HSP27	↑ (2–8 Gy)	↑ PC3, LNCaP, HPrEPiC ↓ DU145	RR	Mimicking	[37]

TS—Tumor suppressor miRNA; OM—Oncogenic miRNAs; RR—Radioresistant; RS—Radiosensitive; -—absent of information; ↑—increased expression; ↓—decreased expression.

**Table 2:** Sets of miRNA primer sequences for RT-qPCR.

MiRNA	Primer Forward
**miR-95**	UUCAACGGGUAUUUAUUGAGCA
**miR-106-5p**	UAAAGUGCUGACAGUGCAGAU
**miR-145-5p**	GUCCAGUUUUCCCAGGAAUCCCU
**miR-541-3p**	UGGUGGGCACAGAAUCUGGACU
**Universal**	Universal PCR Primer
**RNU6**	GUGCUCGCUUCGGCAGCACAUAUACUAAAAUUGGAACGAUACAGAGAAGAUUAGCAUGGCCCCUGCGCAAGGAU-GACACGCAAAUUCGUGAAGCGUUCCAUAUUUU

## Data Availability

All data that support the findings of this study are available within the article or from the corresponding authors upon reasonable request.

## Supplementary Materials

The following supporting information can be downloaded at: https://www.mdpi.com/article/10.3390/ijms25052754/s1.
