# Supplementary material for: Radiotherapy Metastatic Prostate Cancer Cell Lines Treated with Gold Nanorods Modulate miRNA Signatures
Source: Int J Mol Sci. 2024 Feb 27;25(5):2754. doi: 10.3390/ijms25052754 (PMC10931958; doi:10.3390/ijms25052754)
Supplement: Supplementary file 1 [file ijms-25-02754-s001.zip › ijms-2804148-supplementary.pdf]

Supplementary Materials

Radiotherapy Metastatic Prostate Cancer Cell Lines Treated with Gold Nanorods Modulate miRNA Signatures

Sílvia Soares [1,2,3,4,5,6,7], Fátima Aires [7], Armanda Monteiro [7], Gabriela Pinto [7], Isabel Faria [2,3], Goreti Sales [5,6], Miguel A. Correa-Duarte [8,9], Susana Guerreiro [1,10,11,12,†] and Rúben Fernandes [1,2,3,13,†,*]

[1] (i3S), Instituto de Investigação e Inovação em Saúde, 4200-135 Porto, Portugal
[2] FP-I3ID, Instituto de Investigação, Inovação e Desenvolvimento; FP-BHS, Biomedical and Health Sciences, Universidade Fernando Pessoa (UFP), 4249-004 Porto, Portugal
[3] CECLIN, Centro de Estudos Clínicos, Hospital Escola Fernando Pessoa, 4420-096 Gondomar, Portugal
[4] Faculty of Chemistry, University of Vigo, 36310 Vigo, Spain
[5] CEB, Centre of Biological Engineering, Minho University, 4710-057 Braga, Portugal
[6] Biomark@UC/CEB–Centre of Biological Engineering of Minho University, Department of Chemical Engineering, Faculty of Sciences and Technology, Coimbra University, 3030-790 Coimbra, Portugal
[7] Radiotherapy Service, São João Hospital Center, 4200-319 Porto, Portugal
[8] CINBIO, University of Vigo, 36310 Vigo, Spain
[9] Southern Galicia Institute of Health Research (IISGS), Biomedical Research Networking Center for Mental Health (CIBERSAM), 36310 Madrid, Spain
[10] Institute of Molecular Pathology and Immunology of the University of Porto-IPATIMUP, 4200-465 Porto, Portugal
[11] Department of Biomedicine, Biochemistry Unit, Faculty of Medicine, University of Porto, 4200-319 Porto, Portugal
[12] Faculty of Nutrition and Food Sciences, University of Porto, 4150-180 Porto, Portugal
[13] UFP@RISE, Rede de Investigação em Saúde, Universidade Fernando Pessoa, 4249-004 Porto, Portugal
* Correspondence: ruben.fernandes@ufp.edu.pt
† These authors contributed equally to this work.

Supplementary Materials:

Table S1. Expression levels of miRNAs in PC3 prostate cancer cell line. RT-qPCR analysis of miRNAs relative to their levels in control group without any treatment. RNU6 housekeeping gene was used for normalization of expression and gene relative expression was employed by the ΔCT expression/ΔCT control ratio. Regarding irradiations, cells were irradiated in three fraction of 2.5 Gy – 1x2.5 Gy, 2x2.5 Gy, and 3x2.5 Gy were the first, second and third fraction respectively.

Sample	miR-95	miR-106-5p	miR-145-5p	miR-541-3p
**Control 0 Gy**	1.0 ± 0.1	1.0 ± 0.1	1.0 ± 0.5	1.0 ± 0.3
**Control 1x2.5 Gy**	0.3 ± 0.1	6.7 ± 0.3	6.7 ± 0.7	0.2 ± 0.0
**Control 2x2.5 Gy**	2.0 ± 0.4	6.2 ± 0.4	7.3 ± 0.1	1.2 ± 0.3
**Control 3x2.5 Gy**	2.4 ± 0.4	4.5 ± 1.9	5.6 ± 0.9	2.5 ± 0.7
**$AuNP_r$ + 0 Gy**	0.7 ± 0.3	0.9 ± 0.2	0.4 ± 0.1	0.5 ± 0.1
**$AuNP_r$ + 1x2.5 Gy**	27.8 ± 15.0	1.9 ± 1.1	5.7 ± 0.3	16.3 ± 0.8
**$AuNP_r$ + 2x2.5 Gy**	2.9 ± 1.6	4.3 ± 1.8	4.7 ± 3.6	2.2 ± 1.3
**$AuNP_r$ + 3x2.5 Gy**	9.8 ± 5.4	2.2 ± 0.0	2.3 ± 1.3	8.6 ± 1.1

Table S2. Expression levels of miRNAs in DU145 cell line. qPCR analysis of miRNAs relative to their levels in control group without any treatment. RNU6 was used for normalization of expression. Gene relative expression was employed by the ΔCT expression/ΔCT control ratio. Regarding irradiations, cells were irradiated in three fraction of 2.5 Gy – 1x2.5 Gy, 2x2.5 Gy, and 3x2.5 Gy were the first, second and third fraction respectively.

Sample	miR-95	miR-106-5p	miR-145-5p	miR-541-3p
**Control 0 Gy**	1.0 ± 0.3	1.0 ± 0.0	1.2 ± 1.0	1.0 ± 0.1
**Control 1x2.5 Gy**	0.4 ± 0.1	1.1 ± 0.4	1.0 ± 0.3	0.2 ± 0.1
**Control 2x2.5 Gy**	0.2 ± 0.1	0.7 ± 0.1	0.5 ± 0.4	0.1 ± 0.0
**Control 3x2.5 Gy**	1.2 ± 0.8	1.2 ± 0.4	1.6 ± 1.3	0.3 ± 0.1

**AuNP$_r$ + 0 Gy**	0.2 ± 0.1	0.5 ± 0.0	0.4 ± 0.2	0.1 ± 0.0
**AuNP$_r$ + 1x2.5 Gy**	0.2 ± 0.1	0.8 ± 0.0	1.0 ± 0.1	0.1 ± 0.0
**AuNP$_r$ + 2x2.5 Gy**	0.4 ± 0.2	0.5 ± 0.1	0.5 ± 0.1	0.2 ± 0.1
**AuNP$_r$ + 3x2.5 Gy**	0.4 ± 0.2	0.8 ± 0.1	1.1 ± 0.1	0.3 ± 0.0

Table S3. Expression levels of miRNAs in LNCaP cell line was evaluated by qPCR analysis of miRNAs relative to their levels in control group without any treatment. RNU6 was employed for normalization of expression and gene relative expression was used by the ΔCT expression/ΔCT control ratio. Regarding irradiations, cells were irradiated in three fraction of 2.5 Gy – 1x2.5 Gy, 2x2.5 Gy, and 3x2.5 Gy were the first, second and third fraction respectively.

Sample	miR-95	miR-106-5p	miR-145-5p	miR-541-3p
**Control 0 Gy**	1.0 ± 0.4	1.0 ± 0.1	1.0 ± 0.1	1.0 ± 0.3
**Control 1x2.5 Gy**	3.2 ± 4.0	6.4 ± 0.8	3.5 ± 0.6	8.8 ± 0.6
**Control 2x2.5 Gy**	2.6 ± 0.4	1.3 ± 0.1	1.4 ± 0.2	1.3 ± 0.2
**Control 3x2.5 Gy**	3.4 ± 0.1	1.8 ± 0.8	1.8 ± 0.9	2.2 ± 0.4
**AuNP$_r$ + 0 Gy**	1.2 ± 0.6	0.8 ± 0.1	0.7 ± 0.6	1.2 ± 0.4
**AuNP$_r$ + 1x2.5 Gy**	2.2 ± 0.9	1.7 ± 0.0	2.5 ± 0.2	2.4 ± 0.3
**AuNP$_r$ + 2x2.5 Gy**	2.3 ± 0.4	1.3 ± 0.6	1.0 ± 0.4	2.4 ± 1.8
**AuNP$_r$ + 3x2.5 Gy**	1.8 ± 1.1	1.3 ± 0.1	0.8 ± 0.3	1.5 ± 0.0

Table S4. Expression levels of miRNAs in HPrEpiC, non-tumor cell line. qPCR was used to evaluated miRNAs expression and the results were normalized to RNU6. Gene relative expression was used by the ΔCT expression/ΔCT control ratio. Regarding irradiations, cells were irradiated in three fraction of 2.5 Gy – 1x2.5 Gy, 2x2.5 Gy, and 3x2.5 Gy were the first, second and third fraction respectively.

Sample	miR-95	miR-106-5p	miR-145-5p	miR-541-3p
**Control 0 Gy**	1.0 ± 0.3	1.0 ± 0.0	1.2 ± 1.0	1.0 ± 0.5
**Control 1x2.5 Gy**	2.4 ± 0.4	1.9 ± 0.4	1.3 ±1.0	2.5 ± 0.2
**Control 2x2.5 Gy**	4.0 ± 0.8	3.5 ± 0.1	4.3 ± 2.8	1.5 ± 2.0
**Control 3x2.5 Gy**	2.7 ± 0.1	2.2 ± 1.0	1.2 ± 0.1	1.6 ± 0.2
**AuNP$_r$ + 0 Gy**	1.0 ± 0.8	1.0 ± 0.0	0.4 ± 0.1	0.7 ± 0.2
**AuNP$_r$ + 1x2.5 Gy**	3.6 ± 2.1	2.8 ± 0.2	2.8 ± 0.8	2.3 ± 0.4
**AuNP$_r$ + 2x2.5 Gy**	4.2 ± 0.7	2.0 ± 0.6	1.1 ± 0.4	2.1 ± 1.3
**AuNP$_r$ + 3x2.5 Gy**	3.6 ± 2.5	2.7 ± 0.1	2.2 ± 0.4	4.4 ± 0.5
